# Effects of intradialytic aerobic exercise on hemodialysis patients: a systematic review and meta-analysis

**DOI:** 10.1007/s40620-018-00565-z

**Published:** 2019-01-18

**Authors:** Nada Salhab, Mirey Karavetian, Jeroen Kooman, Enrico Fiaccadori, Cosette F. El Khoury

**Affiliations:** 1 School of Nutrition and Translational Research in Metabolism, Faculty of Health Medicine and Life Sciences, Maastricht University, Maastricht, The Netherlands; 2 Department of Health Sciences, College of Natural Health Sciences, Zayed University, Dubai, United Arab Emirates; 3 Department of Internal Medicine, Division of Nephrology, University Hospital Maastricht, Maastricht, The Netherlands; 4 Internal Medicine and Nephrology Department, Parma University Medical School, Parma, Italy; 5 School of Public Health and Primary Care, Faculty of Health Medicine and Life Sciences, Maastricht University, Maastricht, The Netherlands

**Keywords:** Hemodialysis, Intradialytic exercise, Quality of life, Phosphorus, Meta-analysis

## Abstract

**Objective:**

Intradialytic exercise (IDE) is not yet a routine practice for hemodialysis patients, the lack of guidelines supporting it being a major reason. This systematic review and meta-analysis of aerobic IDE interventions examined the efficacy of IDE regarding quality of life (QOL), serum phosphorus, dialysis efficiency, inflammatory status, vitamin D3, parathyroid hormone, intake of phosphate binders, mortality and hospitalization rate.

**Methods:**

Pubmed, Medline (Ovid), Embase (Ovid), Cochrane, and Cinahl (EBSCO) databases were searched to retrieve studies up to June 12, 2018. A manual reference search was also performed. Studies were included if they evaluated (a) aerobic IDE effect on at least one of our study parameters, (b) adult hemodialysis patients, (c) patients for > 1 month.

**Results:**

Twenty-two studies were retrieved (706 participants), of which 12 were eligible for meta-analysis. Aerobic IDE had a significant positive effect on the QOL physical component score (QOL-PSC) and on mental component score (QOL-MCS) of SF36, but not on serum phosphorus or Kt/V.

**Conclusions:**

IDE incorporation into clinical practice has a significant positive effect on QOL-PSC and QOL-MCS. In the reviewed studies, IDE did not result in any health hazard in hemodialysis patients. Nevertheless, future research should assess the long-term effectiveness and safety of IDE. The limitations of this review include the lack of quality analysis of the studies, the limited number of studies that could be included in the meta-analysis, the diversity in the exercise intensity, duration and modality, and the limited data for several outcomes.

**Prospero registration ID:**

CRD42016052062.

## Introduction

Intradialytic exercise (IDE) is defined as exercise training performed during the hemodialysis (HD) session to increase the patient’s strength and endurance, and hence targeting various physiological and psychosocial parameters. The nature of the IDE varies from resistance to aerobic exercise and stretching, with different equipment used corresponding to the type of exercise. IDE has demonstrated a positive effect on the overall health and hospitalization rate of HD patients [1, 2].

The Kidney Disease Improving Global Outcomes (KDIGO) guidelines recommend a full integration of exercise in the daily life of chronic kidney disease patients (at least 30 min/day, 5 times a week) taking into consideration their cardiovascular health and level of tolerance [3]. Unfortunately, there are no clear guidelines for IDE, and the IDE experience is described, from the patient’s perspective, as “going into the unknown” [4].

Sedentary lifestyle, low quality of life and reduced VO_2_ max are associated with increased mortality risk among HD patients [5–7]. IDE has shown to have positive effects in HD patients; this was evident from the results of previous systematic reviews where IDE improved the efficiency of dialysis (Kt/V) [8, 9], VO_2_ max [8–10], and quality of life (QOL) [8–11]. In addition, several studies have also shown positive effects of IDE on solute clearance, notably with regard to serum phosphorus levels (P) [1, 12–14]. Possible explanations of the underlying mechanism are the increased cardiac output and blood flow to lower extremities, and capillary vasodilation, resulting in more solutes being transferred to the vascular compartment and reaching the dialyzer membrane for diffusion [15]. Thus IDE could be a cornerstone in the management of HD patients.

Capitanini et al. suggested that, to sustain an exercise program for HD patients, there needs to be a healthcare team led by a nephrologist and including a cardiologist, physiotherapist, exercise physiologist, renal dietitian, and a nurse [16]. To date, some dialysis units are lucky enough to have ergometers that connect to the HD beds or chairs so permitting their HD patients to benefit from this IDE aerobic exercise. Furthermore, exercise prescription for several chronic conditions [17] is in alignment with the current international physical activity (PA) guidelines which is 150 min/week of moderate intensity aerobic PA [18]. Even though other recommendations include resistance training, this review will focus on the effects of aerobic IDE since combined training is more complex for HD patients.

This review adds serum phosphate (P) as a new parameter (with respect to existing reviews), since it is an important risk factor associated with negative outcomes in HD patients [19]. The objective of this review is to explore all published interventions on aerobic IDE, identify the optimal protocols used, and highlight the effects of these trials on patient QOL, P, dialysis efficiency (urea reduction ratio [URR], Kt/V), vitamin D3, parathyroid hormone (PTH), and serum C-reactive protein (CRP), intake of P binders, the number of emergency HD sessions, cost effectiveness, mortality and hospitalization rate.

## Methods

### Eligibility criteria

All eligible studies were included if they met the predetermined inclusion and exclusion criteria detailed in the PICOS model (Table 1). There was no language restriction, nor filters applied to the search.

**Table 1:** Eligibility criteria

Criteria	Inclusion criteria	Exclusion criteria
Population	Chronic adult HD patients	All other patients
Intervention	Aerobic IDE	Non aerobic exercise
		Non-IDE intervention
Comparison	Pre-post intervention	
Outcomes	Intervention-control group	
	QOL	
	Serum P	
	Kt/v	
	URR	
	Vitamin D3	
	PTH	
	CRP	
	Mortality	
Study types	Hospitalization rate	
	Interventional studies	Interventions < 1 month
		Non-interventional trials
		Conference abstracts
		Systematic reviews
		Meta-analyses

### Search strategy

Potential studies were identified using the Pubmed, Medline (Ovid), Embase (Ovid), Cochrane, and Cinahl (EBSCO) databases up to June 12, 2018. In addition, references of included articles were hand-searched by 2 reviewers; pertinent references were retrieved and submittted to full text screening. The search strategy included the following terms selected from the Medical Subject Headings (MeSH): renal replacement therapy, renal dialysis, hemodiafiltration, hemodialysis/home, renal insufficiency, renal osteodystrophy, dialysis, kidneys/artificial, motor activity, exercise, walking, physical exertion, physical endurance, exercise tolerance, physical fitness, exercise therapy, sports, bicycling, psychomotor performance, motor skills; it also included the following keywords: renal, kidney, insufficien*, replacement*, failure*, osteodytroph*, necrosis, necroses, artificial, dialy*, microdialy*, haemodialy*, hemodialy*, intradialy*, hemodiafiltration*, haemodiafiltration*, hemo-diafiltration*, haemo-diafiltration*, cardio, muscle*, muscular, endurance, aerobic*, physical, training, capacity*, capab*, therap*, toleran*, prescri*, interven*, techni*, physical*, motion*, activit*, fit, fitness, function*, exert*, modalit*, motor, skill*, psychomotor, performance. The asterisk (*) sign denotes that the word before it will be searched in all its possible versions. Boolean operators “OR, AND” were used for the search. The full search strategy is available on https://www.crd.york.ac.uk/PROSPEROFILES/52062_STRATEGY_20180205.pdf.

This systematic review and meta-analysis followed the PRISMA (Preferred Reporting Items for Systematic Reviews and Meta-Analyses) guidelines [20] and was registered on Prospero (registration ID: CRD42016052062; available at: http://www.crd.york.ac.uk/PROSPERO/display_record.php?ID=CRD42016052062). A medical librarian validated the search strategy.

### Study selection

Three reviewers (N.S., M.K., C.K.) independently assessed all retrieved articles. Overall average pairwise percent agreement between the 3 reviewers was 96.667% (Fleiss Kappa coefficient = 0.967; Krippendorff’s Alpha = 0). Only one article was disagreed upon by reviewer #3, and a consensus was reached through discussion between the 3 reviewers.

### Data analysis

Three authors (N.S., M.K., C.K.) developed a template for data extraction; information extracted included patient characteristics, study design, exercise prescription and outcome variables (Kt/v, URR, serum P, QOL, vitamin D3, PTH, CRP, cost effectiveness, hospitalization rate, number of emergency HD, intake of P binders, mortality). Eligible articles were reviewed, and relevant data was then extracted, and organized into 2 main results tables by one author (N.S.).

The meta-analysis was performed using the Explanatory Software for Confidence Intervals (ESCI) program [21], based on the change in P, QOL-PCS, QOL-MCS and Kt/v which is the difference in mean between the start and end of the intervention. Pooled standard deviation was also calculated using the following formula:

$$\surd \left[ {\left( {{\text{SEM pre intervention}}} \right)^{{\text{2}}} + {\text{ }}\left( {{\text{SEM post intervention}}} \right)^{{\text{2}}} } \right]$$

where SEM = SD/√sample size. Heterogeneity was assessed across studies using the diamond ratio (DR); a DR > 2 implies considerable heterogeneity [22]. In the case of heterogeneity, a random-effects model was used. The meta-analysis was conducted for outcomes reported in a minimum of 3 studies, due to the potential for greater uncertainty with fewer studies [23]. The effect size (ES) of controlled studies was calculated, when possible, to evaluate the effectiveness of the IDE intervention. The reported ES was based on the relative size of Cohen’s d, which categorizes the studies in the following four levels: d < 0.2 = negligible effect, over 0.2 = a small effect, over 0.5 = a medium effect, and over 0.8 = a large effect [24]. The corresponding 95th confidence intervals (CIs) were also reported for each outcome.

## Results

**Fig. 1:**
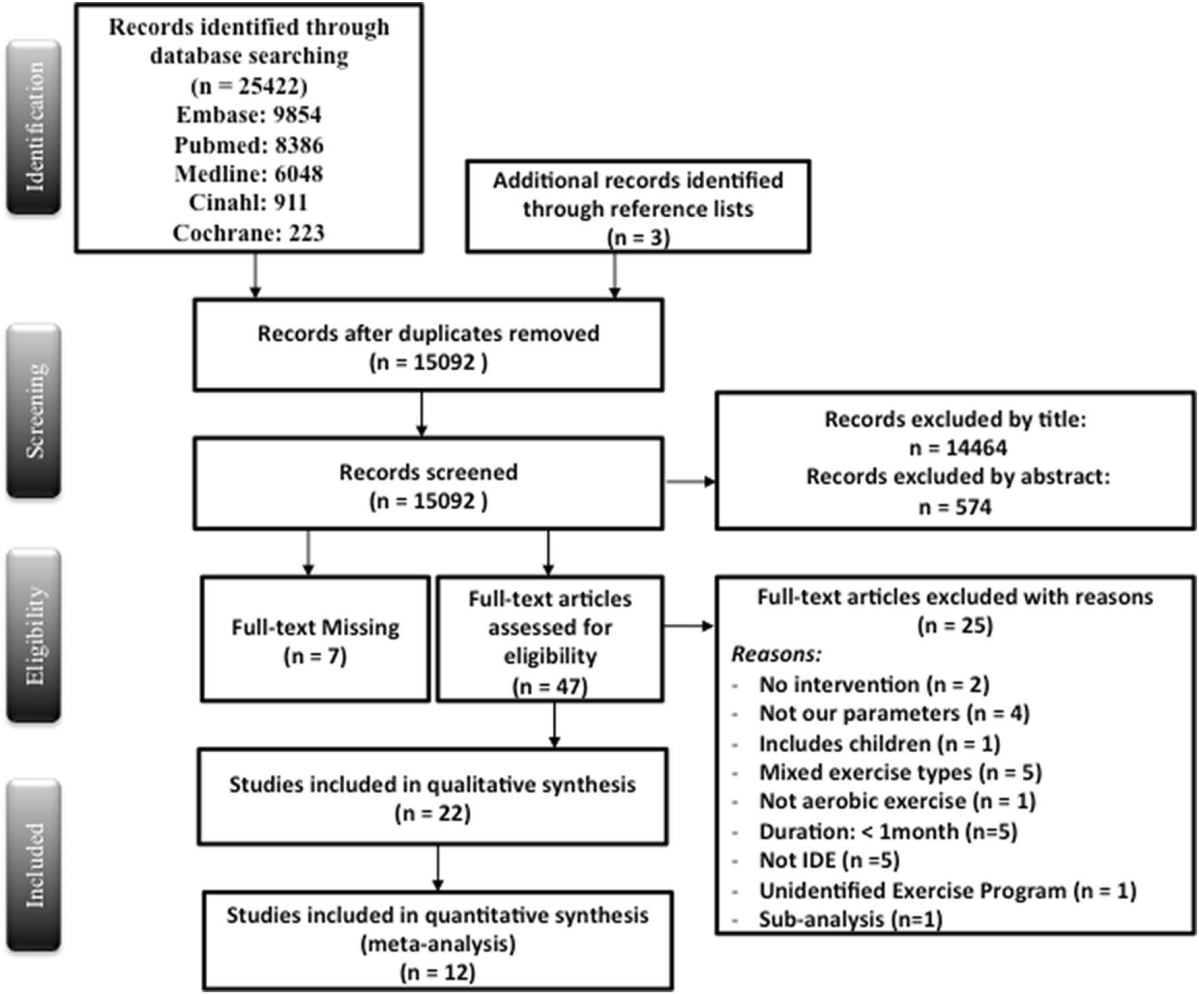
Flow chart of study selection based on the PRISMA statement

This systematic review was able to identify a wide range of parameters, possibly related to a positive effect of IDE, such as Kt/v, P, QOL, URR, hospitalization, and CRP. After title and abstract screening, the searches resulted in 15,092 articles, of which 52 were eligible for full text screening. The authors excluded 25 articles for one of the following reasons: no intervention was conducted, study outcomes were not measured, the study included children, had mixed types of exercise or non-aerobic exercises, had an intervention duration of < 1 month, or the full text could not be retrieved even after contacting the authors. Thus 22 articles were included in this review (Fig. 1). Only the randomized controlled trials (RCTs) and the interventions with comparison groups with common outcomes were included in the meta-analysis (12 studies). The main characteristics of controlled and uncontrolled studies are presented respectively in Tables 2 and 3. The percentage change of all outcomes reported in Tables 2 and 3 was calculated by the authors using the pre and post outcome results using the following formula: ((Post Outcome Result – Pre Outcome Result)/Pre Outcome Result)*100.

**Table 2:** Intradialytic aerobic exercise and quality of life studies

References and country	Sample	Intervention		Outcomes		
		Design	Exercise prescription	Variables + Groups	% Change (Cohen’s d)	*p* value
Wu et al. [25] China	I (n = 32); C (n = 33)	RCT:		I: [QOL-SF36]	+ 37.7%	< 0.05^a,b^
	Age (yrs ± SD)	3 months; 3 times/week	I: 5 min warm up + 10–15 min of cycling Intensity: Borg scale set between 12–16^¶^	C: [QOL-SF36]	+ 2.8%	NS^a^
	I (45/37–48); C: (44/41–50)	20 min/session	C: 10–15 min simple stretching exercises			
	Gender:	Setting: 1 HD unit				
	I: 84.4% M; C: 84.8% M					
De Lima et al. [1] Brazil	I (n = 10); C (n = 11)	RCT:	I: cycling	I: [KDQOL-SF 1.3—physical functioning]	NR	< 0.05^b^
	Age (yrs ± SD)	2 months; 3 times/week	Intensity: Borg scale set between 10–12^¶^	I: [KDQOL-SF 1.3—pain]	NR	< 0.05^b^
	I (43.1 ± 13.3); C (43.5 ± 11.1)	20 min/session	C: Received routine care	I: [KDQOL-SF 1.3—symptoms]	NR	< 0.04^b^
	Gender:	Setting: 1 HD unit		I: [KDQOL-SF 1.3—sleeping]	NR	< 0.006^b^
	I: 50% M; C: 54.5% M			I: [KDQOL-SF 1.3—sexual function]	NR	< 0.01^b^
				I: [KDQOL-SF 1.3—energy/fatigue]	NR	< 0.02^b^
				C: [KDQOL-SF 1.3]	NR	< 0.05^b^
Giannaki et al. [26] Greece	I (n = 15); C (n = 7)	RCT:	I: cycling	I: [QOL-SF36—MCS]	+ 15.2% (0.26)	NS^a^, NS^b^
	Age (yrs ± SD)	6 months; 3 times/week	Intensity: 60–65% of patient’s maximal exercise capacity	C: [QOL-SF36—MCS]	− 4.5%	NS^a^
	I (56.4 ± 12.5); C (56.8 ± 16.5)	45–60 min/session	C: Received routine care + placebo pills	I: [QOL-SF36—PCS]	+ 17.7% (0.27)	0.003^a^, NS^b^
	Gender:	Setting: 1 HD unit		C: [QOL-SF36—PCS]	+ 9.4%	NS^a^
	I: 73.3% M; C: 71.4% M					
Dobsak et al. [27] Czech Republic	I (n = 11); C (n = 10)	RCT:	I: 5 min warm up + 20–40 min cycling + 5 min cool down	I: [QOL-SF36—MCS]	+ 8.5% (0.03)	0.001^a^, NR^b^
	Age (yrs ± SD)	4.6 months; 3 times/week	Intensity: 30–60% of patient’s peak workload	C: [QOL-SF36—MCS]	+ 1.7%	NS^a^
	I (58.2 ± 7.2); C (60.1 ± 8.1)	30–50 min/session	C: Received routine care	I: [QOL-SF36—PCS]	+ 11.1% (0.19)	0.006^a^, NR^b^
	Gender:	Setting: 1 HD unit		C: [QOL-SF36—PCS]	− 1.5%	NS^a^
	I: 36.3% M; C: 66.6% M					
Koh et al. [28] Australia	I (n = 15); C (n = 16)	RCT:	I: 2 weeks of conditioning: 15 min cycling/session	I: [QOL-SF36—MCS]	− 3.3% (0.26)	NS^a^, NS^b^
	Age (yrs ± SD)	5 months; 3 times/week	9 weeks: 30 min cycling/session	C: [QOL-SF36—MCS]	− 3.0%	NS^a^
	I (52.3 ± 10.9); C (51.3 ± 14.4)	15 to 45 min/session	9 weeks: 45 min cycling/session	I: [QOL-SF36—PCS]	− 11.3% (0.35)	NS^a^, NS^b^
	Gender:	Setting: 3 HD units	Intensity: Borg scale set between 12–13^¶^	C: [QOL-SF36—PCS]	No Δ	NS^a^
	I: 66.6% M; C: 50%		C: Received routine care			
Sakkas et al. [29] Greece	I (n = 7); C (n = 7)	2 Groups intervention:	I: 5 min warm up + 45 min cycling + 5 min cool down	I: [QOL-SF36—MCS]	+ 26.6% (0.99)	NS^a^, NR^b^
	Age (yrs ± SD)	3.7 months; 3 times/week	Intensity: 65–75% of patient’s maximum power capacity	C: [QOL-SF36—MCS]	− 10.1	NS^a^
	I (48 ± 14); C (70 ± 11)	55 min/session	C: Received routine care	I: [QOL-SF36—PCS]	+ 23.8% (1.78)	0.01^a^, NR^b^
	Gender:	Setting: 1 HD unit		C: [QOL-SF36—PCS]	+ 14.5%	NS^a^
	I: 71.4% M; C: 71.4% M					
Parsons et al. [30] Canada	I (n = 6); C (n = 7)	RCT:	I: three 15 min exercise bouts of cycling	I: [QOL-SF36—physical function]	+ 1.1%	NS^a^, NR^b^
	Age (yrs ± SD)	2 months; 3 times/week	Intensity: 40–50% of maximal work capacity	C: [QOL-SF36—physical function]	− 4.2%	NS^a^
	I (60 ± 17); C (49 ± 25)	45 min/session	C: Received routine care	I: [QOL-SF36—role emotional]	− 7.7%	NS^a^, NR^b^
	Gender:	Setting: 1 HD unit		C: [QOL-SF36—role emotional]	+ 17.6%	NS^a^
	I: 50% M; C: 57.1% M			I: [QOL-SF36—bodily pain]	+ 2.1%	NS^a^, NR^b^
				C: [QOL-SF36—bodily pain]	+ 10.1%	NS^a^
				I: [QOL-SF36—general health]	− 13.7%	NS^a^, NR^b^
				C: [QOL-SF36—general health]	− 13.4%	NS^a^
				I: [QOL-SF36—vitality]	− 6.9%	NS^a^, NR^b^
				C: [QOL-SF36—vitality]	+ 1.2%	NS^a^
				I: [QOL-SF36—social function]	− 2.6%	NS^a^, NR^b^
				C: [QOL-SF36—social function]	+ 12.4%	NS^a^
				I: [QOL-SF36—role physical]	+ 16.4%	NS^a^, NR^b^
				C: [QOL-SF36—role physical]	+ 26.7%	NS^a^
				I: [QOL-SF36—mental health]	+ 3.4%	NS^a^, NR^b^
				C: [QOL-SF36—mental health]	+ 1.0%	NS^a^
Painter et al. [31] USA Painter et al. [31] USA^¥^	I (n = 166); C (n = 28)	2 Groups intervention:	I: strengthening + stretching exercises at home for 2 months followed by intradialytic cycling for 2 months	I: [QOL-SF36—MCS]	+ 4.7% (0.0)	NR^a^, NS^b^
	Age (yrs ± SD)	2 months; 30 min/session		C: [QOL-SF36—MCS]	− 0.1%	NR^a^
	I (55.9 ± 15.15); C (52.8 ± 16.8)	Setting: 5 HD units	Intensity: based on the perceived exertion scale to fit their tolerable rate	I: [QOL-SF36 -PCS]	+ 9.1% (0.64)	NR^a^, 0.001^b^
	Gender:			C: [QOL-SF36—PCS]	− 15.6%	NR^a^
	I: 42.9% M; C: 34.6% M		C: Received routine care			
Chigira et al. [32] Japan	n = 7	Single group Intervention:	15 min of warm up and stretching + 20 min cycling + 1 min cool down	[WHO-QOL26]	+ 4.1%	NS
	Age (yrs ± SD)	3 months; 2–3 times/week 40 min/session	Intensity: Borg scale set between 11 and 13^¶^			
	Gender: 71.4% M	Setting: 1 HD unit				
Bae et al. [33] Korea	n = 10	Single group Intervention:	5 min warm up + 30 min cycling + 2 min cool down	I: [QOL-SF36]	+ 14.7%	NS
	Age (yrs ± SD)	3 months; 2–3 times/week	Intensity: at 35 rpm + Borg scale set between 11 and 13^¶^			
	56.5 ± 4.4	30 min/session				
	Gender: 40% M	Setting: 1 HD unit				
Musavian et al. [14] Iran	n = 16	Single group Intervention:	(a) 8 weeks: control period	(d) [QOL-SF36]	+ 20.8%	0.007
	Age (yrs ± SD)	4 non-consecutive months	(b) 8 weeks: 30 min of passive cycling			
	51.9 ± 1.57	3 times/week	(c) 8 weeks: washout			
	Gender: 81.2% M	30 min/session	(d) 8 weeks: 30 min of active cycling			
		Setting: 1 HD unit	Intensity: not reported			
Bohm et al. [34] Canada	n = 20	Single group Intervention:	Cycling intensity: Borg scale set between 12 and 14^¶^	[QOL-SF36- PCS]	+ 11.4%*	NS
	Age (yrs ± SD)	6 months; 3 times/week		[QOL-SF36- MCS]	− 2.0%*	NS
	52 ± 14.5	60 min/session				
	Gender: 73% M	Setting: 4 HD units				
Golebiowski et al. [35] Poland	n = 21	Single group Intervention:	Cycling intensity: physical load was individually adapted to exercise tolerance	[QOL- SF36v2]	Increase	NS
	Age (yrs ± SD)	3 months; 3 times/week		Sub group analysis of patients with the		
	64.2 ± 13.1	50 ± 19 min/session		lowest [physical function] initially: [physical functioning]	+ 88.8%	0.04
	Gender: 51.7% M	Setting: 1 HD unit				
Besnier et al. [42] France	n = 6	Single group Intervention	Cycling at a speed of 60–70 turns per minute”	[QOL-SF36—PCS]	+ 12.3%	NR
	Age (yrs ± SD)	3 months; 3 times/week	Intensity: Moderate intensity exercise was maintained when patient reaches the value of his first ventilator threshold	[QOL-SF36—MCS]	− 2.8%	NR
	72.5 ± 7.8	30 min/session		Significance unreported		
	Gender: 66.6% M	Setting: 1 HD unit				
Reboredo et al. [13] Brazil	n = 14	Single group Intervention	Pre intervention conditioning: 12 weeks of stretching for 10 min of the lower limbs	[QOL-SF36—physical function]	+ 17.1%	< 0.05
	Age (yrs ± SD)	3 month; 3 times/wee		[QOL-SF36—social functioning]	+ 15.1%	< 0.05
	47.6 ± 12.8	30 min/session	Intervention: 15 min of stretching and warm up + 30 min of cycling + 15 min cool down and stretching	[QOL-SF36—mental health]	+ 18.1%	< 0.05
	Gender: 28.6% M	Setting: 1 HD unit		[QOL-SF36—role physical]	+ 13.4%	NS
			Intensity: Borg scale set between 11–13^¶^	[QOL-SF36—pain]	+ 22.2%	NS
				[QOL-SF36—general health]	− 6.4%	NS
				[QOL-SF36—vitality]	+ 4.1%	NS
				[QOL-SF36—role emotional]	+ 18.0%	NS
McMurray et al. [36] Australia	n = 19	Single group Intervention:	Cycling Intensity: based on individual’s capacity to cope with the program	[QOL-SF12—MCS]	− 12.6%	NS
	Age (yrs ± SD)	3 months; 3 times/week		[QOL-SF12—PCS]	− 22.2%	NS
	67.6 ± 11.2	40 min/session				
	Gender: 63.6% M	Setting: 1 HD unit				
Parsons et al. [37] Canada	n = 13	Single group Intervention:	Cycling: two 30 min exercise bouts with a 30 min recovery period between bouts	[KDQOL]	NR	NS
	Age (yrs ± SD)	5 months; 3 times/week		[QOL-SF36]	NR	NS
	53.0 ± 18.0	60 min/session	Intensity: based on patient’s capacity			
	Gender: 61.5% M	Setting: 2 HD units				

Order of the studies: randomized controlled studies followed by single group studies, and in descending order of the publication year

*p* significance, *Yrs* years, *SD* standard deviation, *M* male, *RCT* randomized controlled trial, *Min* minutes, *HD* hemodialysis, *I* intervention, *C* control, *QOL* quality of life, *NS* non-significant, *NR* not reported, *PCS* physical component summary, *MCS* mental component summary, *USA* United States of America

^¶^Borg scale set between 12–14 is described a “somewhat hard” and is equivalent to a moderate intensity exercise; ^a^significance for within group comparison with baseline; ^b^significance of intervention compared with control group; ¥ this study was a subgroup study so we only reported the results of the mother study; *measurement reported from a figure

**Table 3:** Intradialytic aerobic exercise and biochemical/other parameters studies

References and country	Sample	Intervention		Outcomes		
		Design	Exercise prescription	Variables + Groups	% Change (Cohen’s d)	*p* value
***Dialysis efficiency***						
Groussard et al. [38]	I (n = 8); C (n = 10)	RCT:	I: cycling	I: [Kt/v]	+ 8.3% (0.95)	NR^a,b^
France	Age (yrs ± SD)	3 months; 3 times/week	Intensity: 55–60% of the peak power output	C:[Kt/v]	− 10.2%	NR^a,b^
	I (66.5 ± 4.6); C (68.4 ± 3.7)	40 min/session	C: Received routine care			
	Gender:	Setting: 2 HD units				
	I: 62.5% M; C: 77.7% M					
Dobsak et al. [27]	I (n = 11); C (n = 10)	RCT:	I: 5 min warm up + 20–40 min cycling + 5 min cool down	I: [Kt/v]	+ 14.6% (1.01)	0.02^a^, NR^b^
Czech Republic	Age (yrs ± SD)	4.6 months; 3 times/week	Intensity: 30–60% of patient’s peak workload	C:[Kt/v]	− 5.6%	NS^a^, NR^b^
	I (58.2 ± 7.2); C (60.1 ± 8.1)	30–50 min/session	C: Received routine care	I : [URR %]	+ 10.9% (1.44)	0.001^a^, NR^b^
	Gender:	Setting: 1 HD unit		C: [URR %]	− 3.3%	NS^a^
	I: 36.3% M; C: 66.6% M					
Afshar et al. [39]	I (n = 7); C (n = 7)	RCT:	I: 5 min warm up + 10–30 min cycling	I: [Kt/v]	No **(**0.34)	NS^a^, NR^b^
Iran	Age (yrs ± SD)	2 months; 3 times/week	Intensity: Borg scale set between 12–16^¶^	C: [Kt/v]	+ 0.9%	NS^a^
	I (50.7 ± 21.06); C (53 ± 19.4)	10–30 min/session	C: Received routine care			
	Gender:100% M	Setting: 1 HD unit				
Sakkas et al. [29]	I (n = 7); C (n = 7)	2 Groups intervention:	I: 5 min warm up + 45 min cycling + 5 min cool down	I: [Kt/v]	+ 8.3% (0.22)	NS^a^, NR^b^
Greece	Age (yrs ± SD)	3.7 months; 3 times/week	Intensity: 65–75% of patient’s maximum power capacity	C:[Kt/v]	+ 30%	0.05
	I (48 ± 14); C (70 ± 11)	55 min/session	C: Received routine care			
	Gender:	Setting: 1 HD unit				
	I: 71.4% M; C: 71.4% M					
Parsons et al. [30]	I (n = 6); C (n = 7)	RCT:	I: three 15 min exercise bouts of cycling	I: [Kt/v]	+ 0.7% (0.18)	NS^a^, NR^b^
Canada	Age (yrs ± SD)	2 months; 3 times/week	Intensity: 40–50% of maximal work capacity	C: [Kt/v]	− 3.1%	NS^a^, NR^b^
	I (60 ± 17); C (49 ± 25)	45 min/session	C: Received routine care			
	Gender:	Setting: 1 HD unit				
	I: 50% M; C: 57.1% M					
Chigira et al. [32]	n = 7	Single group Intervention	15 min of warm up and stretching + 20 min cycling + 1 min cool down	[Kt/v]	+ 6.6%	NS
Japan	Age (yrs ± SD)	3 months; 2–3 times/week				
	70.6 ± 4.4	40 min/session	Intensity: Borg scale set between 11 and 13^¶^			
	Gender: 71.4% M	Setting: 1 HD unit				
Musavian et al. [14]	n = 16	Single group Intervention:	(a) 8 weeks: control period	(b) [Kt/v]	+ 8.3%	NS
Iran	Age (yrs ± SD)	2 non-consecutive months	(b) 8 weeks: 30 min of passive cycling	(d) [Kt/v]	− 4.3%	NS
	51.9 ± 1.5	3 times/week	(c) 8 weeks: washout	(b) [URR %]	+ 1.9%	NS
	Gender: 81.2% M	30 min/session	(d) 8 weeks: 30 min of active cycling	(d) [URR %]	− 3.3%	NS
		Setting: 1 HD unit	Intensity: not reported			
Parker et al. [2]	n = 102	Single group Intervention	HD units had established intradialytic bicycling program as part of their routine care	[URR %]	+ 1.4%	0.02
Canada	Age (yrs ± SD)	6 months; 3 times/week				
	65.6 ± 13.5	≥ 30 min/session	Intensity: Borg scale set between 12–13^¶^			
	Gender: 67.6% M	Setting: 2 HD units				
Reboredo et al. [13]	n = 14	Single group Intervention:	Pre intervention conditioning: 12 weeks of stretching for 10 min of the lower limbs	[Kt/V]	+ 41.6%	< 0.05
Brazil	Age (yrs ± SD)	3 months; 3 times/week				
	47.6 ± 12.8	30 min/session	Intervention: 15 min of stretching and warm up + 30 min of cycling + 15 min cool down and stretching			
	Gender: 28.6% M	Setting: 1 HD unit				
			Intensity: Borg scale set between 11–13^¶^			
Parsons et al. [37]	n = 13	Single group Intervention:	Cycling: two 30 min exercise bouts with a 30 min recovery period between bouts	[Kt/v]	+ 15.4%	< 0.05
Canada	Age (yrs ± SD)	5 months; 3 times/week		[URR %]	+ 7.1%	< 0.05
	53.0 ± 18.0	60 min/session	Intensity: based on patient’s capacity			
	Gender: 61.5% M	Setting: 2 HD units				
***Phosphorus***						
De Lima et al. [1]	I (n = 10); C (n = 11)	RCT:	I: cycling	I: [P mg/dl]	− 3.5% (0.14)	NS^a^
Brazil	Age (yrs ± SD)	2 months; 3 times/week	Intensity: Borg scale set between 10–12^¶^	C: [P mg/dl]	+ 3.7%	NS^a^
	I (43.1 ± 13.3); C (43.5 ± 11.1)	20 min/session	C: Received routine care			
	Gender:	Setting: 1 HD unit				
	I: 50% M; C: 54.5% M					
Makhlough et al. [12]	I (n = 25); C (n = 23)	RCT:	I: Aerobic movement exercise of range capacity	I: [P mg/dl]	− 24.0% (0.56)	0.003^a^
Iran	Age (yrs ± SD)	2 months; 3 times/week	Intensity: based on patient’s capacity	C: [P mg/dl]	+ 0.5%	NS^a^, NR^b^
	I (53.3 ± 14.27); C (56.16 ± 10.77)	15 min/ session	C: Received routine care			
	Gender:	Setting: 1 HD unit				
	I: 73.9% M; C: 54.2% M					
Wilund et al. [40]	I (n = 8); C (n = 9)	RCT:	I: cycling	I: [P mg/dl]	+ 25% (0.9)	NS^a^, NR^b^
USA	Age (yrs ± SD)	4 months; 3 times/week	Intensity: Borg scale set between 12–14^¶^	C: [P mg/dl]	− 6.3%	NS^a^, NR^b^
	I (60.8 ± 3.2); C (59.0 ± 4.9)	45 min/session				
	Gender:	Setting: 1 HD unit	C: Received routine care			
	I: 37.5% M; C: 42.8% M					
Musavian et al. [14]	n = 16	Single-group Intervention:	(a) 8 weeks: control period	(b) [P mg/dl]	− 0.4%	NS
Iran	Age (yrs ± SD)	2 non-consecutive months	(b) 8 weeks: 30 min of passive cycling	(d) [P mg/dl]	− 13.1%	0.004
	51.9 ± 1.57	3 times/week	(c) 8 weeks: washout			
	Gender: 81.2% M	30 min/session	(d) 8 weeks: 30 min of active cycling			
		Setting: 1 HD unit	Intensity: not reported			
Reboredo et al. [13]	n = 14	Single-group Intervention	Pre intervention conditioning: 12 weeks of stretching for 10 min	[P mg/dl]	− 7.0%	NS
Brazil	Age (yrs ± SD)	3 months	of the lower limbs			
	47.6 ± 12.8	3 times/week	Intervention: 15 min of stretching and warm up + 30 min of cycling + 15 min cool down and stretching			
	Gender: 28.6% M	30 min/session				
		Setting: 1 HD unit	Intensity: Borg scale set between 11–13^¶^			
McMurray et al. [36]	n = 19	Single-group Intervention:	Cycling	[P mmol/l]	− 2.7%	NS
Australia	Age (yrs ± SD)	3 months; 3 times/week	Intensity: based on individual’s capacity to cope with the program			
	67.6 ± 11.2	40 min/session				
	Gender: 63.6% M	Setting: 1 HD unit				
***Others***						
Afshar et al. [39]	I (n = 7); C (n = 7)	RCT:	I: 5 min warm up + 10–30 min cycling	I: [hs-CRP mg/l]	− 83.8%	Sig^a^, 0.005^b^
Iran	Age (yrs ± SD)	2 months; 3 times/week	Intensity: Borg scale set between 12–16^¶^	C: [hs-CRP mg/l]	+ 1.4%	NS^a^
	I (50.7 ± 21.06); C (53 ± 19.4)	10–30 min/session	C: Received routine care			
	Gender: 100% M	Setting: 1 HD unit				
Wilund et al. [40]	I (n = 8); C (n = 9)	RCT:	I: cycling	I: CRP (mg/dl)	− 5.7%	NS^a^, NR^b^
USA	Age (yrs ± SD)	4 months; 3 times/week	Intensity: Borg scale set between 12–14^¶^	C: CRP (mg/dl)	− 3.2%	NS^a^, NR^b^
	I (60.8 ± 3.2); C (59.0 ± 4.9)	45 min/session				
	Gender:	Setting: 1 HD unit	C: Received routine care			
	I: 37.5% M; C: 42.8% M					
Golebiowski et al. [35]	n = 21	Single-group Intervention:	Cycling	[CRP mg/l]	+ 15.0%	NS
Poland	Age (yrs ± SD)	3 months; 3 times/week	Intensity: physical load was individually adapted to exercise tolerance			
	64.2 ± 13.1	50 ± 19 min/session				
	Gender: 51.7% M	Setting: 1 HD unit				
Parker et al. [2]	n = 102	Single-group Intervention	HD units had established intradialytic bicycling program as part of their routine care	[Hospitalization rate]	− 2.6%	NS
Canada	Age (yrs ± SD)	(Retrospective Study)				
	65.6 ± 13.5	6 months; 3 times/week	Intensity: Borg scale set between 12–13^¶^			
	Gender: 67.6% M	≥ 30 min/session				
		Setting: 2 HD units				

Order of the studies: randomized controlled studies followed by single group studies, and in descending order of the publication year

*p* significance, *Yrs* years, *SD* standard deviation, *M* male, *RCT* randomized controlled trial, *Min* minutes, *HD* hemodialysis, *I* intervention, *C* control, *Sig* significant, *NS* non-significant, *NR* not reported, *P* phosphorus, *URR* urea reduction ration, *PRU* percentage reduction ration, *CRP* C-reactive protein

^a^significance for within group comparison with baseline; ^b^significance of intervention compared with control group; ^¶^Borg scale set between 12–14 is described a “somewhat hard” and is equivalent to a moderate intensity exercise

### General specifications of studies

The number of participants in a single study ranged from 6 to 194, and the mean age of participants varied between 43.3 and 72.5 years. Male gender was predominant in most studies except for 5 [13, 27, 31, 33, 40, 41]. The IDE in 13 studies consisted of cycling only [1, 2, 14, 26, 28, 30, 34, 36–40, 42], and 8 studies included warm-up and stretching in addition to cycling [13, 25, 27, 29, 31–33, 35]. One study consisted of aerobic range of motion capacity only [12]. Exercise intensity was assessed in 11 studies [1, 2, 13, 25, 28, 31–34, 39, 40] using the Borg Rating of Perceived Exertion Scale, set between 10 and 16. The other studies used either the patient’s maximal exercise capacity, ranging between 30 and 75% of oxygen consumption capacity (VO_2_ max) [26, 27, 29, 37, 38] or the patient’s individual capacity (4 studies) [12, 35–37]. One study did not report the intensity [14] and another one set the cycle speed at 60–70 revs/min [39]. Single IDE sessions varied in duration between 15 and 60 min, a duration of ≥ 30 min being the most used one. As for the length of the interventions, it varied between 2 and 6 months. IDE was performed at every HD session (2 or 3 times per week).

### IDE effects on quality of life

A total of 17 studies [1, 13, 14, 25–37, 42] showed the effect of aerobic IDE on QOL. Data were very heterogeneous due to the use of different QOL instruments. Accordingly, a meta-analysis including all studies could not be conducted. Table 2 shows detailed data from each study.

A total of 5 studies were included in the meta-analysis [26–29, 31] and reported the QOL—physical component scale (PCS) (Table 4) and the QOL—mental component scale (MCS) (Table 4), in a total number of 282 patients (214 patients in the experimental group and 68 patients in the control group). Results showed considerable heterogeneity. Overall, our meta-analysis indicated that aerobic IDE had a significant positive effect on both the QOL-PCS and QOL-MCS. In our meta-analysis, the study with large effect size for the QOL-PCS and the QOL-MCS had the youngest patients in the intervention group and the oldest in the control group [29]. The study with medium effect size for the QOL-PCS was that of Painter et al. in which the methodology used was radically different compared to the other studies, i.e. there were 2 months of conditioning exercises at home prior to the 2 months of IDE [31]. Moreover, the positive effect of IDE preconditioning on the QOL was also seen in the single arm interventional study conducted by Musavian et al. [14].

**Table 4:**
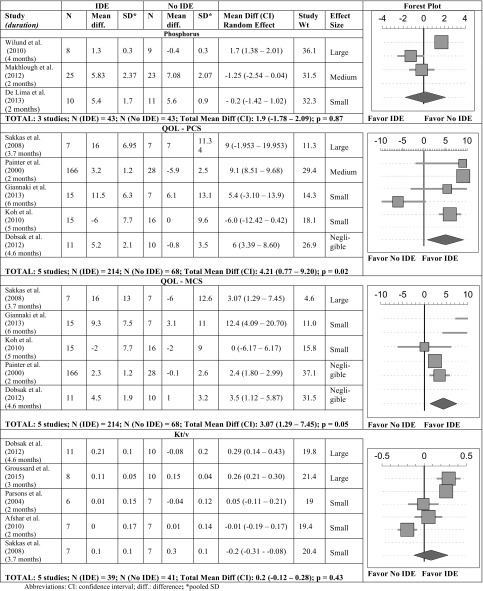
Forest plot of a random effect meta-analysis assessing the change in QOL-PCS, QOL-MSC, Kt/v and P in IDE interventions versus controls

As for the total QOL score, the most significant percentage change was reported in an RCT by Wu et al. (37.7%) where the design included 5 min warm-up, and 20 min cycling exercise for a period of 3 months at an intensity of 12–16 points on the Borg scale [25].

Also, two single-group interventions [14, 33] had a high percentage change in the total QOL-SF-36 score: 20.8% [14] and 14.7% [33]. These latter two studies had a similar IDE program to Wu et al. [25] except that they had a longer duration exercise session.

### IDE effects on phosphorus

Six studies [1, 12–14, 36, 40] assessed the effect of IDE on serum phosphorus (P) level. Only 3 were eligible to be included in the meta-analysis. They showed considerable heterogeneity, and indicated that IDE had no significant effect on serum P levels (Table 4). Makhlough et al. reported the highest positive effect size and was the only to show a significant P decrease [12]; their intervention consisted of aerobic range of motion capacity for 15 min/session for 2 months. This study was also unique in the fact of having the highest mean baseline serum P (7.68 mg/dl) for the IDE group compared to the other studies included in this systematic review. Contrary to the other 2 RCTs [1, 12], Wilund et al. showed an increase in the P level post IDE; the study had the most prolonged intervention period (4 months) and exercise duration (45 min), yet the lowest sample size; the mean starting value for serum P in the intervention group was 5.2 mg/dl [40].

As for the other 3 single-group interventions [13, 14, 36], serum P levels were lower at the end of the study, the decrease being statistically significant only in the shortest duration intervention [14], where patients exercised for 30 min/session (but the intensity was not reported). It is worth noting that the 3 studies all started with a P level ranging between 4.82 and 5.7 mg/dl.

### IDE effects on dialysis efficiency

Ten studies assessed the effect of IDE on Kt/V [13, 14, 27, 29, 30, 32, 37–39] and URR [2, 14, 27, 37]. Only 5 were eligible for the meta-analysis [27, 29–31, 38], and they showed considerable heterogeneity. Their pooled analysis showed that IDE had no significant effect on Kt/V (Table 4). Nevertheless, Dobsak et al. was able to show a large effect size and a positive change (+ 14.6%) on Kt/V [27]. This study was unique in its gender distribution (63.7% of the intervention group were females), duration (4.6 months), intensity (30–60%), and session length (30–50 min). Another study conducted by Groussard et al. had a large effect size with a positive change (+ 8.3%) on Kt/v [38]. Two other studies also reported a positive change in Kt/v post intervention but had a small effect size [29, 30].

Among the single-group interventions, the 2 studies that showed the most significant change in Kt/v were those by Reboredo et al. and Parsons et al. [13, 37]; the former study included 3 months IDE preconditioning before the intervention and the latter one had the longest IDE duration per session and intervention length. Concerning URR, one RCT showed a large effect size and reported a positive change (+ 10.9%) in URR post intervention [27]; the single-group intervention study of Parsons et al. also showed a positive URR change and had the longest session length (60 min) [37].

### Effect of IDE on inflammatory status variables

Three studies assessed the effect of IDE on CRP [35, 39, 40]. Only 2 were RCTs [39, 40] and showed a decrease in CRP level contrary to the single-group pilot study [35] where CRP increased but not significantly. Afshar et al.’s study showed the most significant change and was unique in its gender distribution, being 100% male [39]. A meta-analysis was not possible due to the low number of eligible studies reporting on CRP.

### Effect of IDE on hospitalizations

Hospitalization rate was reported in only 1 single-group intervention [2], and showed a non-significant decrease over 6 months.

### Effect of IDE on other outcomes

None of the articles identified through our screening methodology measured vitamin D3, PTH, cost effectiveness, number of emergency HD, intake of P binders, and mortality, which were initially planned to be assessed in this systematic review. Thus, we cannot report on these parameters.

## Discussion

This systematic review focused on the effect of aerobic IDE programs in an HD setting. The conclusions drawn from this review are not only based on the meta-analysis results, since some of our main outcome parameters were trialed only in studies without comparators.

A meta-analysis of 5 articles showed that aerobic IDE improves both QOL-PCS and QOL-MCS. This is partly in line with findings reported in the literature, where the meta-analysis of studies on aerobic and resistance IDE programs showed that IDE had a significant difference on the QOL-PCS but not on the QOL-MCS [8, 10]. Pre-IDE muscle conditioning and young age were identified as the key components of success in terms of patients being able to maintain a long-term IDE program and thus harvest the positive clinical outcomes [14, 31]. However, a recent review conducted by Gomes et al. showed that aerobic IDE was not associated with improvement in the QOL [43]. However, Gomes et al.’s meta-analysis included an article in which IDE targeted malnourished patients; thus it has different selection criteria and its findings cannot be generalized to the general HD population. Our review, however, included 1 article with a large sample size that might have affected the results. In addition, our meta-analysis was based on the outcome change rather than on post intervention values.

In our review we could not identify an overall effect of IDE on P levels in HD patients. However, a possible effective IDE recipe to improve serum P was identified by Makhlough et al. [12] where patients included in the study were hyperphosphatemic (serum P > 4.5 mg/dl) [44] and the exercise involved was a 2-month aerobic exercise program involving range of motion capacity. Moreover, Musavian et al. had an added value of preconditioning exercises before implementation of IDE programs on their serum P results [14]. These results may be explained by the fact that hyperphosphatemic patients are the ones that need additional therapies to improve their current status, thus they would be the ones that reap the effect of IDE the most [14, 44]. Out of the 3 RCTs focusing on serum P, two of them had an increase in P in their control groups, thus we postulate that the IDE program reversed the natural deterioration of serum P commonly seen among HD patients; this gives more importance to the positive clinical effect of IDE. HD patients have numerous comorbidities reducing the magnitude of improvement [31]; this very fact may be more obvious when the parameter taken into account is initially in the normal range. Wilund et al. had the highest exercise duration and length of intervention, but the P level increased at the end of the intervention [40]. The authors did not define the mechanisms of this increase and mentioned their small sample size as a limitation, preventing control for various factors. In addition, Wilund et al. did not report if dietary P was monitored.

Our review did not find an overall positive effect of IDE on Kt/v in HD patients. On the other hand, time seems to be the key for Kt/V improvement with IDE. When comparing the 5 RCTs, characterized by similar duration of exercise per dialysis session and intensity, the most prolonged exercise program [27] resulted in the most significant change. Also, gender may play a role since the only intervention that showed no change was in a study on male patients only. Among the single-group interventions, Reboredo et al. showed the largest positive significant change (+ 41.6%) in Kt/V [13]; this study had the youngest patients, and the highest female gender percentage among the other comparable studies [14, 32, 37]. It included conditioning before intervention and stretching along with cycling. Another meta-analysis [8] showed that IDE groups had higher Kt/v values than the control group; however, the type of exercise included also resistance and stretching [31, 45, 46], not only aerobic exercise. Unlike Sheng et al., we were not able to identify a clear effect of aerobic IDE on Kt/V. Thus we cannot exclude that other exercise modalities could be more beneficial.

There was no association between IDE and mortality risk or rates of hospitalization in the included studies; this was also reported in a previous systematic review [11] where no RCT on exercise training with chronic kidney disease patients reported exercise as a direct cause of death. Nephrologists should capitalize on this fact to advocate exercise to their patients. Nevertheless, data insufficiency in this field may be a major source of hesitation in advocating IDE.

Other factors beyond those studied in the present paper may provide arguments in favor of IDE. After the initiation of HD therapy, patients’ lean tissue mass tends to decrease and fat tissue mass and body mass index (BMI) tend to increase [47], which may contribute to sarcopenic obesity [48]. Fluctuation of fat and lean tissue is affected by gender, comorbidities and the initial body composition [47]. Thus, assessing the body composition in HD patients is crucial, especially since BMI is not always a good indicator in this population [49]. Mortality is higher in HD patients with a low fat tissue index (FTI) and lean tissue index (LTI) [49]; whereas survival is higher in patients with fat and lean tissue compartments lying between the 10th and 90th percentile of a healthy population [49]. It has also been shown that physical inactivity is, in combination with a decline in lean tissue, related to muscle weakness [50] which is an important determinant of outcome in dialysis patients [51]. Whereas resistance-based IDE was shown to increase muscle strength and quadriceps cross-sectional area, its effect on LTI was less apparent, this in contrast to treatment with anabolic steroids [52]. Therefore, assessing body composition as well as muscle strength on a routine basis may be useful for both risk stratification as well as targeted interventions.

Cardiovascular diseases are a major comorbidity in HD patients [53]. National and international guidelines for cardiac rehabilitation include education, exercise training and psychological support [54] as part of their programs. HD patients are mostly cardiac patients; therefore, like the cardiologist, nephrologists should be encouraged to focus on their patient’s behavioral goals through counseling and follow-ups.

HD patients spend an average of 12 h weekly being sedentary on dialysis; thus it is a good opportunity to integrate IDE. IDE might not be a miraculous cure, but it could surely add more functionality to the time spent in HD, improve patients’ QOL, decrease anxiety, and maybe increase adherence to the treatment. Perhaps, IDE could be coupled with music, since music therapy in HD patients has shown it can reduce anxiety [55], pain and nausea [56], while it improves blood pressure, quality of sleep, fever, cramps, anxiety and depression levels, pain, and itching [57].

Based on the studies that showed statistically significant positive effects of IDE on all study parameters in our review, a putative recipe for aerobic exercise in chronic hemodialysis patients could be suggested (Table 5). Exercise, specifically intradialytic, should be part of the HD treatment protocol. Guidelines are required for IDE to be an adjunct therapy to HD.

**Table 5:** Putative recipe for aerobic IDE

Preconditioning	Strengthening and stretching exercise 2 months prior to exercise initiation
Modality	Warm up: Range capacity exercise + Cycling
Frequency	3 times per week
Intensity	Borg scale average rating of 12 points
	Or 60–65% of the peak power output
Duration	Range capacity exercises: 15 min + Cycling: 30–45 min

### Limitations

The limitations of this review were the lack of quality analysis of the studies. As for the general limitations related to the available published literature, these ranged from the selection of the healthier HD patients, to the scarcity of the available interventions, to the limited number of eligible interventions for meta-analysis, to the diversity in exercise intensity, duration and modality across the studies. Furthermore, the presence of a publication bias in this new researched field cannot be underestimated. The authors could not report on all the primary and secondary outcomes that they had planned to evaluate because some of these were unavailable in the selected studies.

## Conclusions

In conclusion, the results of this systematic review suggest that aerobic IDE did not impose any health hazard in HD patients and its incorporation into clinical practice can result in significant improvement in QOL-PCS and QOL-MCS [26–29, 31]. Since sarcopenic obesity is a prevalent phenomenon among HD patients, this can be a call for making IDE a routine practice in HD units. But the accurate mode of delivery (intensity, type, methods) needs to be tailored to each subgroup of patients within the context of the country, culture, and healthcare system.

Future research should assess the long-term effectiveness and safety of IDE. Most of the studies were conducted in Western countries and thus more insights are needed concerning other regions, such as the Middle East. Last but not least, the mortality rate related to IDE should be investigated in order to strengthen the rationale for IDE programs.
